# N6‐methyladenosine‐modified circTEAD1 stabilizes Yap1 mRNA to promote chordoma tumorigenesis

**DOI:** 10.1002/ctm2.1658

**Published:** 2024-04-24

**Authors:** Hanwen Li, Yingchuang Tang, Xingbang Ruan, Junxin Zhang, Hao Liu, Shiyu Yu, Hao Chen, Huilin Yang, Kai Zhang, Kangwu Chen

**Affiliations:** ^1^ Department of Orthopedic Surgery The First Affiliated Hospital of Soochow University Suzhou People's Republic of China; ^2^ Institute of Translational Medicine, Medical College Yangzhou University Yangzhou People's Republic of China

**Keywords:** Chordoma, CircRNAs, Hippo pathway, N6‐methyladenosine modification

## Abstract

**Background:**

Chordoma, a rare bone tumour with aggressive local invasion and high recurrence rate with limited understanding of its molecular mechanisms. Circular RNAs (circRNAs) have been extensively implicated in tumorigenesis, yet their involvement in chordoma remains largely unexplored. N6‐methyladenosine (m6A) modification holds a crucial function in regulating protein translation, RNA degradation and transcription.

**Methods:**

Initially, screening and validation of circTEAD1 in chordoma were conducted by high‐throughput sequencing. Subsequently, sh‐circTEAD1 and an overexpression plasmid were constructed. Colony formation assays, cell counting kit‐8, Transwell and wound healing assays were utilized to validate the function of circTEAD1 in vitro. RNA pull‐down assays identified the binding proteins of circTEAD1, which underwent verification through RNA immunoprecipitation (RIP). Methylated RIP assays were conducted to detect the m6A binding sites. Following this, luciferase assay, RT‐qPCR, RIP and Western blotting analyses were conducted, revealing that Yap1 was the direct target of circTEAD1. Afterwards, the same methods were utilized for the validation of the function of Yap1 in chordoma in vitro. Finally, the regulatory relationship between circTEAD1 and Yap1 in chordoma was verified by an in vivo tumour formation assay.

**Results:**

CircTEAD1 was identified as an upregulated circRNA in chordoma specimens, with heightened circTEAD1 expression emerging as a prognostic indicator. In vitro experiments convincingly demonstrated that circTEAD1 significantly promoted chordoma cell invasion, migration and aggressiveness. Furthermore, the analysis revealed that methyltransferase‐like 3‐mediated m6A modification facilitated the cytoplasmic export of circTEAD1. The circTEAD1/IGF2BP3/Yap1 mRNA RNA‐protein ternary complex not only bolstered the stability of Yap1 mRNA but also exerted a pivotal role in driving chordoma tumorigenesis.

**Conclusions:**

In this study, the role of m6A‐modified circTEAD1 in chordoma was identified. The findings offer novel insights into the potential molecular targets for chordoma therapy, shedding light on the intricate interplay between circRNAs, m6A modification and Yap1 mRNA in chordoma pathogenesis.

## Graphical abstract

**Highlights**:

1. CircTEAD1 is an upregulated circRNA that serves as a prognostic indicator in chordoma.
2. The export of circTEAD1 to the cytoplasm is mediated by METTL3 in an m6A methylation‐dependent manner.
3. CircTEAD1 enhances Yap1 mRNA stability by forming a circTEAD1/IGF2BP3/Yap1 mRNA ternary complex, which leads to chordoma invasion and proliferation.

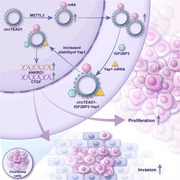

## 1 INTRODUCTION

Chordomas are exceedingly rare malignant tumours originating from embryologic notochord remnants in the axial skeleton.^1^, ^2^ Surgical resection remains the primary treatment modality, often complemented by radiation therapy,^3^ considering the ineffectiveness of conventional chemotherapeutic agents.^4^ The molecular mechanisms of chordomas remain elusive due to their low occurrence.^5^ While clinical studies on molecular targeted therapy have identified potential therapeutic targets in chordoma progression,^6^ currently available drugs have proven ineffective.^3^ Thus, it is necessary to comprehensively explore the molecular mechanisms driving the development of chordoma in order to determine potential targets for therapeutic interventions.

Circular RNAs (circRNAs) have been recognized as a diverse group of primarily non‐coding RNAs (ncRNAs),^7^ capable of regulating a wide array of cellular processes.^8^ Recent research has demonstrated their significant influence on the onset and progression of cancer, employing various mechanisms of action.^9^ CircRNAs have been demonstrated to function as miRNA sponges, thereby repressing miRNA function,^10^, ^11^ and also to interact with RNA‐binding proteins (RBPs).^12^ Despite these intriguing findings, critical circRNA abnormalities, their functions and underlying mechanisms in chordoma remain largely unexplored.

N6‐methyladenosine (m6A), a prevalent RNA modification occurring at the N6‐position of adenosine, represents the most prevalent modification found in ncNRAs and mRNAs.^13^ M6A modification plays a pivotal role in directing RNA degradation, transcription and protein translation.^14^ Alterations in m6A levels have been reported in various cancer pathogenesis and development processes,^15^ with significant implications for circRNAs. Although Lin et al. summarized the progress of m6A‐modification and circRNAs in the regulation of tumour resistance,^16^ further comprehensive and in‐depth exploration is required to understand their biological effects. Moreover, the impact of m6A‐modified circRNAs on chordoma is largely uncharted. Considering this, high‐throughput sequencing was employed to identify relevant circRNAs and delve deeper into the mechanisms driving chordoma development.

In this research, circTEAD1, a specific circRNA that demonstrates significant abundance in human chordoma tissues, was isolated, leading to the exploration of its oncogenic role in chordoma pathogenesis. Initially, the involvement of circTEAD1 in promoting chordoma cell invasion and migration was validated. Additionally, it was revealed that methyltransferase‐like 3 (METTL3) regulates circTEAD1, facilitating its entry into the cytoplasm to function. Significantly, elevated cytoplasmic circTEAD1 promotes the stabilization of Yap1 mRNA by interacting with the RBP Insulin‐Like Growth Factor 2 mRNA‐Binding Protein 3 (IGF2BP3), forming circTEAD1/IGF2BP3/Yap1 mRNA ternary complex. In addition, the impact of Yap1 mRNA on the proliferative and invasive capacities of chordoma was demonstrated. CircTEAD1 regulates the Hippo signalling pathway by promoting the expression of Yap1 mRNA, thereby fuelling the aggressive nature of chordoma cells. The findings propose circTEAD1 as a potential marker for detecting chordoma tumorigenesis and as a promising therapeutic target against this challenging malignancy.

## 2 RESULTS

### 2.1 Screening and features of deregulated circTEAD1 in chordoma tissues

**FIGURE 1:**
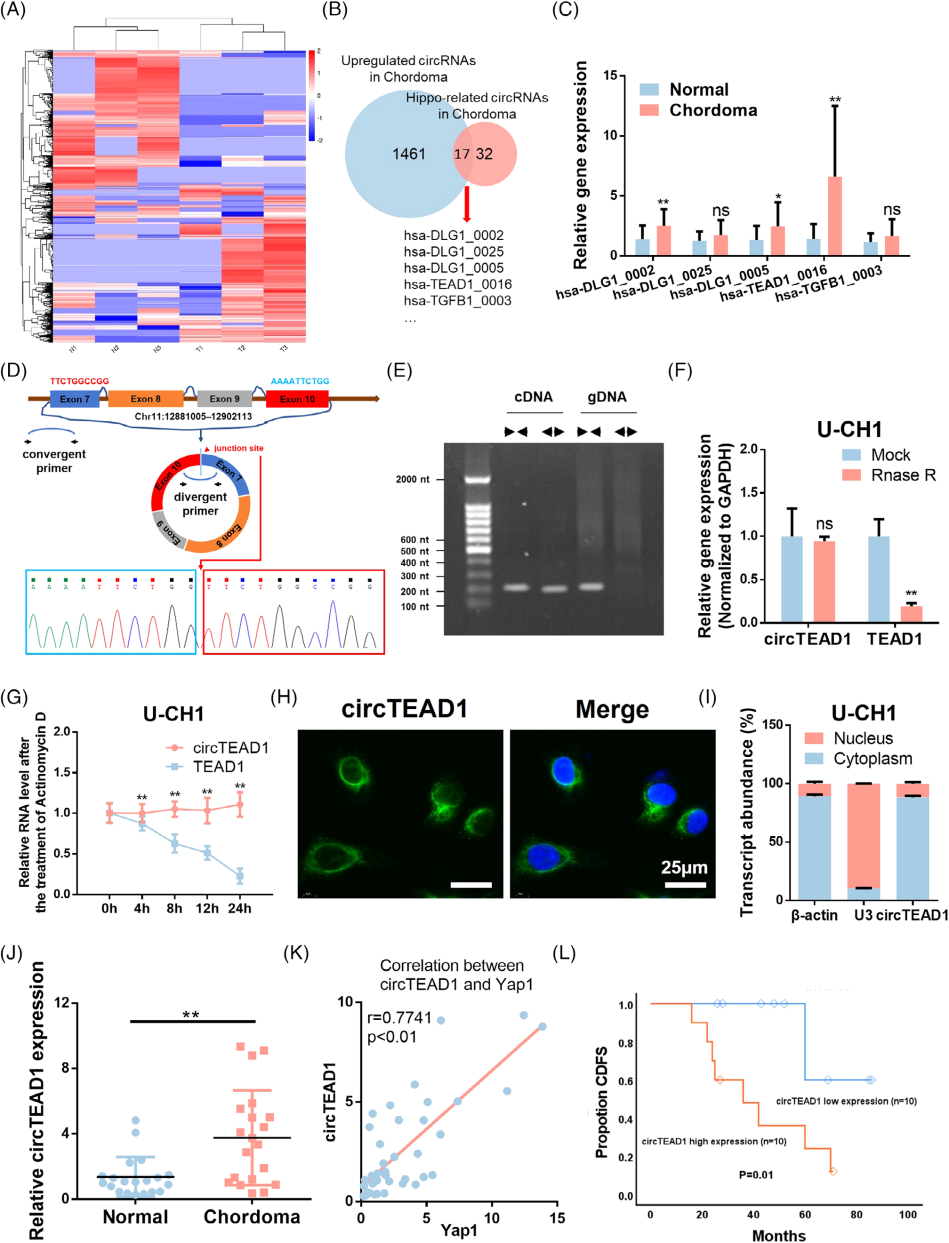
Screening and characteristics of deregulated circTEAD1 in chordoma tissues. (A) The heatmap for dysregulated circRNAs in normal and chordoma tissues. (B) The Venn chart shows the overlap of upregulated circRNAs in chordoma and Hippo‐pathway‐related circRNAs. (C) RT‐qPCR analysis for the top five upregulated circRNAs related to Hippo pathway in normal and chordoma tissues. (D) The genomic location of circTEAD1. Backsplicing in exon7 and exon10 led to the junction of circTEAD1, which was confirmed by the Sanger sequencing. (E) Agarose gel analysis of PCR production using circTEAD1 divergent primer and convergent primer. (F) RT‐qPCR analysis for the expressions of circTEAD1 and TEAD1 treated with RNase R in U‐CH1 cells. (G) RT‐qPCR analysis for the expressions of circTEAD1 and TEAD1 treated with actinomycin D in U‐CH1 cells after 0, 4, 8, 12 and 24 h. (H) RNA fluorescence in situ hybridization (FISH) for circTEAD1 in U‐CH1 cells. Nuclei were stained with DAPI. (I) RT‐qPCR analysis for cytoplasmic and nuclear mRNA fractionation experiment. β‐Actin and U3 were applied as positive controls in the cytoplasm and nucleus, respectively. (J) RT‐qPCR analysis of circTEAD1 expression in normal and chordoma tissues. (K) Correlation plot of relative circTEAD1 and Yap1 mRNA expression. (L) Continuous disease‐free survival (CDFS) according to expression of circTEAD1 in chordoma.

To investigate the dynamics of circRNA alteration within chordoma tissues, high‐throughput circRNA sequencing was conducted utilizing three pairs of chordoma samples, along with their matched adjacent normal tissue counterparts. Notably, a total of 1461 upregulated circRNAs were identified in chordoma tissues (Figure 1A). Brachyury has been shown to regulate the invasiveness of chordoma by controlling the synthesis and stability of Yes‐associated protein (YAP), a key transcription factor in the Hippo pathway.^17^ Therefore, 17 upregulated circRNAs associated with the Hippo pathway (Figure 1B, path: hsa04390) were identified. Subsequently, RT‐qPCR was employed to validate the relative gene expression (Figure S1A). Among the top five circRNAs, hsa‐circ_TEAD1_0016 (circTEAD1) exhibited the most pronounced and consistent alteration (Figure 1C). Consequently, it was selected for further investigation, characterized as circTEAD1, originating from the TEAD1 linear gene, with a total length of 283 bp.

CircTEAD1 is composed of four consecutive exons, and the head‐tail junction connects exons 7 and 10. The circTEAD1 sequence, especially around the junction site, underwent validation via Sanger sequencing (Figure 1D). Subsequent analysis indicated that circTEAD1 is predominantly present as cDNA instead of gDNA (Figure 1E). Moreover, experiments involving RNase R digestion and actinomycin D RNA stability assays were conducted in chordoma cells (U‐CH1/U‐CH2). Compared to its parental gene, circTEAD1 displayed remarkable resistance to RNase R digestion, underscoring its stability attributed to its circular nature (Figure 1F and Figure S1B). Furthermore, upon actinomycin D treatment of chordoma cells, circTEAD1 exhibited a significantly longer half‐life compared to that of TEAD1 (Figure 1G and Figure S1C). The aforementioned findings collectively confirm the robust structural stability of circTEAD1. Nuclear/cytosolic RNA fractionation was conducted in two chordoma cells to guarantee the cellular localization of circTEAD1, with subsequent RT‐qPCR analyses. This suggests that circTEAD1 is mainly distributed in the cytoplasm (Figure 1I and Figure S1D), as further verified by fluorescence in situ hybridization (FISH) assays (Figure 1H and Figure S1E).

To validate the expression levels of circTEAD1 in chordoma tissues, RT‐qPCR was conducted on 20 chordoma tissue samples and 20 matched adjacent normal tissues. The results indicated significantly higher circTEAD1 expression in chordoma tissues in comparison to adjacent normal ones (Figure 1J). Meanwhile, the expression of Yap1 mRNA in clinical samples and its correlation with the expression of circTEAD1 were examined. As a result, a remarkable relationship between the expression of Yap1 and circTEAD1 was found (Figure 1K). Additionally, high circTEAD1 expression in chordoma tissues exhibited a significant correlation with continuous disease‐free survival (CDFS). Notably, our observations indicated that low circTEAD1 expression is associated with longer CDFS in chordoma patients (Figure 1L). Collectively, these results underscore the prevalent and generally elevated expression of circTEAD1 in chordoma tissues, while also highlighting its association with prognosis.

### 2.2 CircTEAD1 promotes the invasive and migratory abilities of chordoma cells

**FIGURE 2:**
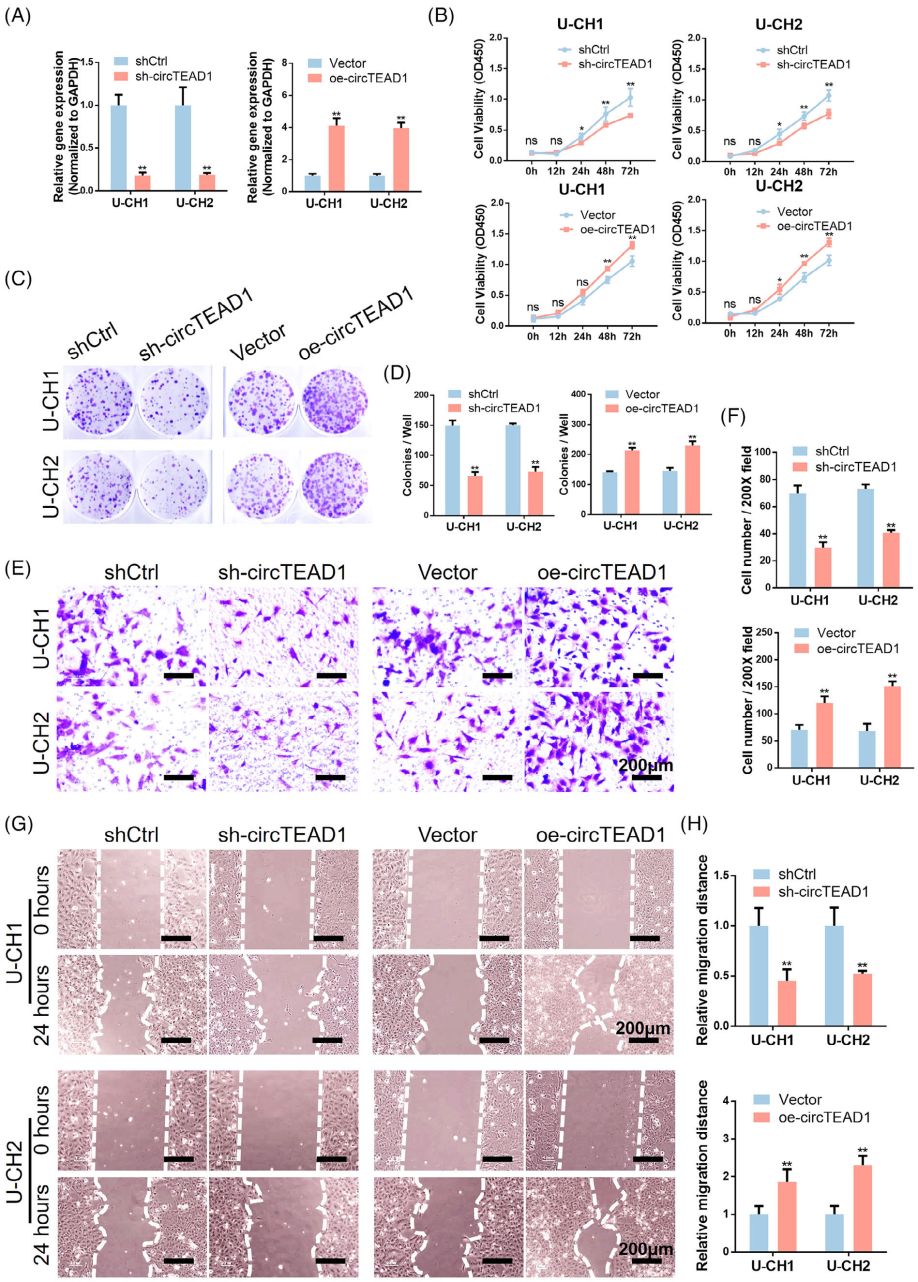
circTEAD1 promotes chordoma cell invasion and migration. (A) RT‐qPCR analysis of knockdown and overexpression efficiency in U‐CH1/U‐CH2 cells transfected sh‐circTEAD1 and oe‐circTEAD1 compared to shCtrl and Vector, respectively. (B) CCK‐8 assays of knockdown and overexpression of circTEAD1 in U‐CH1/U‐CH2 cells. (C) Colony formation assays of knockdown and overexpression of circTEAD1 in U‐CH1/U‐CH2 cells. (D) Quantitative analysis of colony formation assays in (C). (E) Transwell assays of knockdown and overexpression of circTEAD1 in U‐CH1/U‐CH2 cells. (F) Quantitative analysis of Transwell assays in (E). (G) Wound‐healing assays of knockdown and overexpression of circTEAD1 in U‐CH1/U‐CH2 cells. (H) Quantitative analysis of wound‐healing assays in (G).

To examine the role of circTEAD1 in chordoma cells, shRNAs (sh‐circTEAD1, the control is shCtrl) and an overexpression plasmid (oe‐circTEAD1, the control is Vector) were designed and synthesized for transfecting chordoma cells. As anticipated, the RT‐qPCR findings clearly indicated that sh‐circTEAD1 markedly reduced circTEAD1 expression, whereas oe‐circTEAD1 significantly elevated circTEAD1 expression (Figure 2A). By utilizing the cell counting kit‐8 (CCK‐8), it was observed that circTEAD1 substantially promoted the proliferative capacity of chordoma cells (Figure 2B), and consistent results were observed in colony formation assays (Figure 2C,D). Subsequent Transwell assays revealed a remarkable decrease in the number of migrated and invaded cells following circTEAD1 inhibition (Figure 2E,F). Moreover, the results of the wound healing assay revealed a decrease in migration area after 24 h in the sh‐circTEAD1 group, whereas overexpression of circTEAD1 accelerated migration (Figure 2G,H). Collectively, these outcomes emphasize the involvement of circTEAD1 in enhancing the invasion, migration and overall aggressiveness of chordoma cells.

### 2.3 METTL3 facilitates cytoplasmic export of m6A‐modified circTEAD1 for functional activity

**FIGURE 3:**
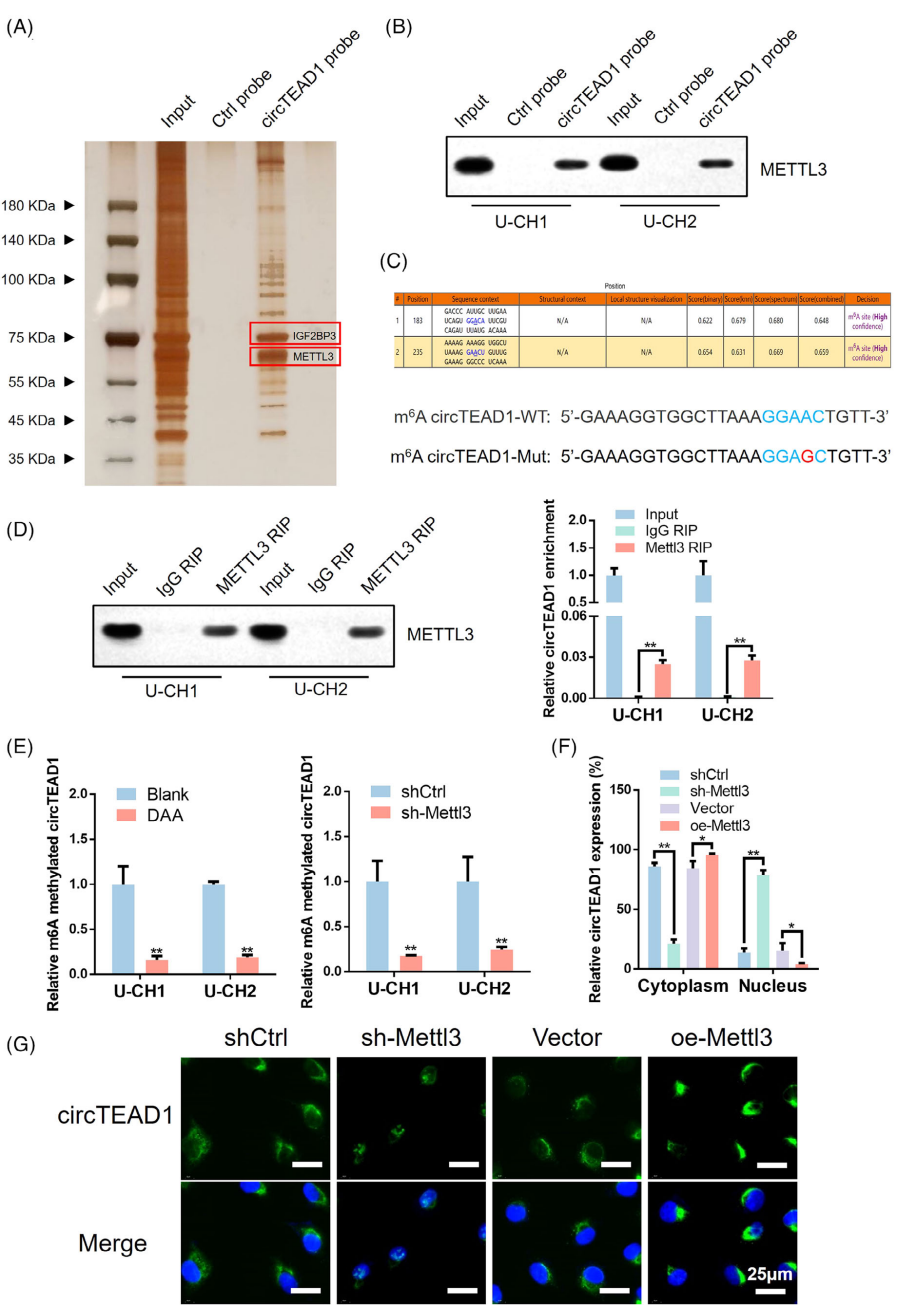
METTL3 facilitates cytoplasmic export of m6A‐modified circTEAD1 for functional activity. (A) Identification of the circTEAD1‐protein complex pulled down by circTEAD1 junction probe with protein extracts from chondroma cells. (B) Immunoblot analysis of METTL3 after pull‐down assay showing its specific association with circTEAD1. (C) Potential m6A sites of circTEAD1 were predicted via the online tool SRAMP. (D) RIP assays showing the association of METTL3 with circTEAD1. Left, IP efficiency of METTL3‐antibody shown in Western blotting. Right, relative circTEAD1 enrichment. (E) MeRIP assay showing that circTEAD1 was highly recruited in m6A precipitated fraction induced by METTL3. DAA mediated total methylation inhibition. (F) RT‐qPCR of cytoplasmic and nuclear mRNA fractionation experiment after U‐CH1 treated with knockdown or expression of Mettl3. (G) RNA‐FISH of circTEAD1 in U‐CH1 after treated with knockdown or expression of Mettl3.

For the exploration of the potential molecular mechanisms governing the involvement of circTEAD1 in the regulation of chordoma malignancy, RNA pull‐down assays were initiated, followed by mass spectrometry (MS) analysis to determine proteins interacting with circTEAD1 (Figure 3A,B). This screening identified METTL3 and IGF2BP3 as putative circTEAD1‐binding proteins. Notably, METTL3 is a methylase for m6A. Two high‐confidence m6A modification sites within circTEAD1 were predicted via the online tool SRAMP. Subsequently, the m6A motif in the RNA probe was mutated for methylated RNA immunoprecipitation (MeRIP) assays (Figure 3C). Simultaneously, validation through RT‐qPCR confirmed that the mutated circTEAD1 exhibited reduced cytoplasmic translocation (Figure S2A). Interestingly, it was observed that overexpression of the mutated circTEAD1 failed to promote chordoma cell proliferation and invasive ability (Figure S2B−D). Next, RNA immunoprecipitation (RIP) analysis showed that circTEAD1 was enriched in the METTL3 group (Figure 3D). Silencing METTL3 or inhibiting total methylation with 3‐Deazaadenosine (DAA) resulted in reduced m6A‐methylated circTEAD1, thereby confirming the interaction of METTL3 with circTEAD1 through the m6A‐binding motif (Figure 3E). Moreover, the changes in the intracellular distribution of circTEAD1 were investigated. The cellular localization experiments, including nuclear and cytoplasmic fractionation (Figure 3F and Figure S3A) and FISH analysis (Figure 3G and Figure S3B), revealed that the knockdown of METTL3 elevated the nuclear proportion of circTEAD1, while Mettl3 overexpression exhibited the opposite effect.

### 2.4 Interaction of circTEAD1 with IGF2BP3

**FIGURE 4:**
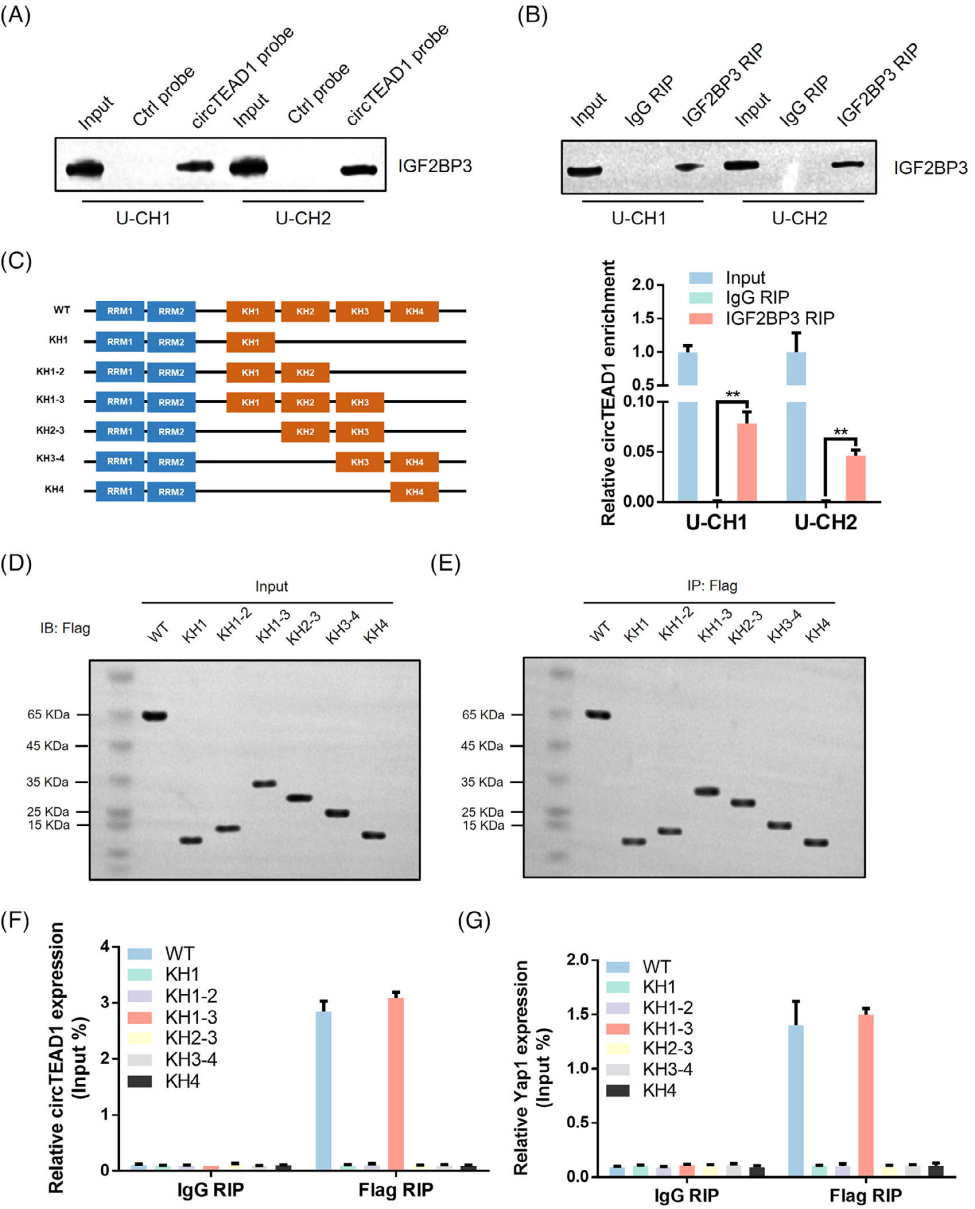
Interaction of circTEAD1 with IGF2BP3. (A) Immunoblot analysis of IGF2BP3 after RNA‐pulldown assay showing its specific association with circTEAD1. (B) RIP assays showing the association of IGF2BP3 with circTEAD1. Relative enrichment representing RNA levels associated with IGF2BP3 compared to an input control. IgG antibody served as a control. (C) Schematic structures showing RNA‐binding domains within IGF2BP3 protein and a summary of IGF2BP3 truncations. (D) Relative circTEAD1 enrichment associated with truncated IGF2BP3 relative to an input control. (E) Immunoblot analysis with anti‐FLAG of U‐CH1 cells transfected with plasmids encoding FLAG‐tagged wild type (WT) or truncated IGF2BP3. (F) RT‐qPCR analysis of circTEAD1 levels associated with truncated IGF2BP3 relative to an input control. (G) RT‐qPCR analysis of Yap1 mRNA levels associated with truncated IGF2BP3 relative to an input control.

Our initial observations from RNA pull‐down assays revealed a robust interaction between circTEAD1 and the abundantly expressed IGF2BP3 protein (Figure 3A and Figure 4A). Furthermore, the RIP experiments revealed an interaction between IGF2BP3 and circTEAD1 (Figure 4B). The objective was to determine which domain of IGF2BP3 is involved in the interaction with circTEAD1 by creating IGF2BP3 mutants with truncations of specific KH domains (Figure 4C−E). Additional RIP assays identified the KH1‐3 tri‐domain of IGF2BP3 as the specific binding site for circTEAD1, underscoring its pivotal role in this interaction (Figure 4F). Based upon earlier identification of circTEAD1 from Hippo‐related circRNAs, the investigation was extended to explore whether Yap1, a key player in the Hippo pathway, also interacts with IGF2BP3. Intriguingly, both circTEAD1 and Yap1 mRNA exhibited a shared preference for binding to the KH1‐3 tri‐domain of IGF2BP3 (Figure 4G). In summary, the findings indicate that circTEAD1 and Yap1 mRNA independently interact with IGF2BP3 within the KH1‐3 tri‐domain.

### 2.5 Formation of the circTEAD1/IGF2BP3/Yap1 mRNA ternary complex stabilizes Yap1 mRNA

**FIGURE 5:**
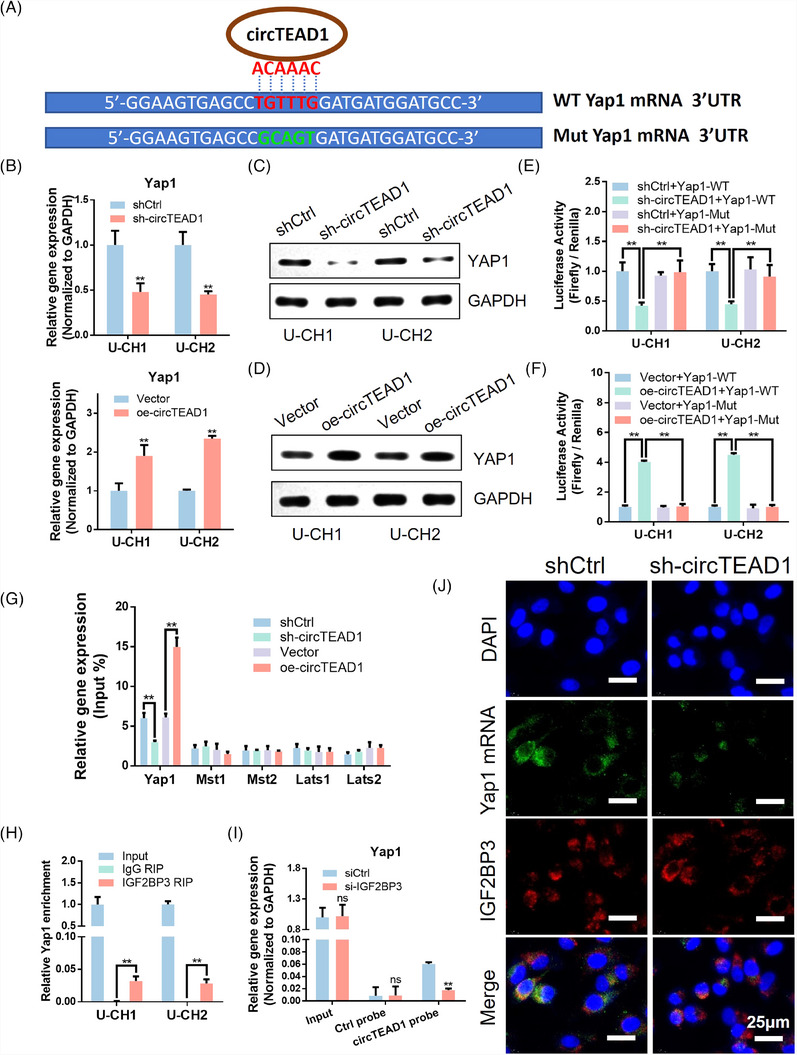
Formation of the circTEAD1/IGF2BP3/Yap1 mRNA ternary complex stabilizing Yap1 mRNA. (A) Sequence BLAST analysis showed that circTEAD1 directly targets the Yap1 mRNA. (B) RT‐qPCR analysis of knockdown and overexpression circTEAD1 in U‐CH1/U‐CH2 cells transfected sh‐circTEAD1 and oe‐circTEAD1 compared to shCtrl and Vector, respectively. (C) Western blotting (WB) analysis of knockdown circTEAD1 in U‐CH1/U‐CH2 cells transfected sh‐circTEAD1 compared to shCtrl. (D) WB analysis of overexpression circTEAD1 in U‐CH1/U‐CH2 cells transfected oe‐circTEAD1 compared to Vector. (E) Relative luciferase activity of luciferase reporter gene with Yap1‐WT or Yap1‐Mut in control and circTEAD1‐knockdown U‐CH1/U‐CH2 cells. (F) Relative luciferase activity of luciferase reporter gene with Yap1‐WT or Yap1‐Mut in control and circTEAD1‐overexpression U‐CH1/U‐CH2 cells. (G) RIP assays showing the association of Yap1 and upstream targets of Hippo‐pathway with circTEAD1 in U‐CH1 cells. (H) RIP assays showing the association of IGF2BP3 with Yap1. (I) RIP assay showing the association of IGF2BP3 with Yap1 in IGF2BP3‐knockdown U‐CH1 cells. (J) Immunofluorescence co‐staining of Yap1 mRNA‐FISH and IGF2BP3 in U‐CH1 cells transfected sh‐circTEAD1 compared to shCtrl. Nuclei were stained with DAPI.

To ascertain whether Yap1 serves as the target of circTEAD1, a sequence BLAST analysis was conducted, revealing that the ACAAAC site within circTEAD1 directly binds to Yap1 mRNA (Figure 5A). Subsequently, both RT‐qPCR and Western blotting analysis substantiated Yap1 as the direct target of circTEAD1 (Figure 5B−D and Figure S4A). The knockdown of circTEAD1 led to a remarkable reduction in the enrichment of Yap1, adding to a corresponding decrease in YAP1 protein expression. Conversely, overexpression of circTEAD1 resulted in the opposite effect. Then, mutations were introduced into the circTEAD1‐binding site within Yap1 mRNA for luciferase reporter minigenes. The knockdown of circTEAD1 significantly suppressed the luciferase mRNA expression and luciferase activity of Yap1‐WT, while having no effect on Yap1‐Mut (Figure 5E and Figure S4B). In contrast, the heightened expression of circTEAD1 markedly elevated both the luciferase mRNA levels and luciferase activity of Yap1‐WT, while again, no impact was detected on Yap1‐Mut (Figure 5F and Figure S4C). Furthermore, RIP was employed to examine whether circTEAD1 interacts with the upstream target of Yap1 in the Hippo pathway (Figure 5G and Figure S4D). The results firmly establish Yap1 as the specific target of circTEAD1 in the Hippo pathway.

Further investigation unveiled that circTEAD1/IGF2BP3/Yap1 mRNA formed an RNA‐protein ternary complex. Firstly, consistent with our earlier RIP assays confirming the interaction of IGF2BP3 with circTEAD1 through the KH1‐3 tri‐domain (Figure 4), it was revealed that IGF2BP3 also interacts with Yap1 (Figure 5H). Knockdown of IGF2BP3 resulted in a reduction in the binding affinity between circTEAD1 and Yap1 mRNA (Figure 5I and Figure S4E). Finally, immunofluorescence revealed the colonization of Yap1 mRNA and IGF2BP3 in the cytoplasm. Notably, the knockdown of circTEAD1 reduced the expression of Yap1, while leaving IGF2BP3 expression unaltered (Figure 5J).

Collectively, these outcomes demonstrate the critical function of circTEAD1 in facilitating interactions between IGF2BP3 and Yap1 mRNA, consequently enhancing the Yap1 mRNA stability through the formation of a circTEAD1/IGF2BP3/Yap1 mRNA RNA‐protein ternary complex.

### 2.6 Yap1 promotes proliferation and invasion in chordoma

**FIGURE 6:**
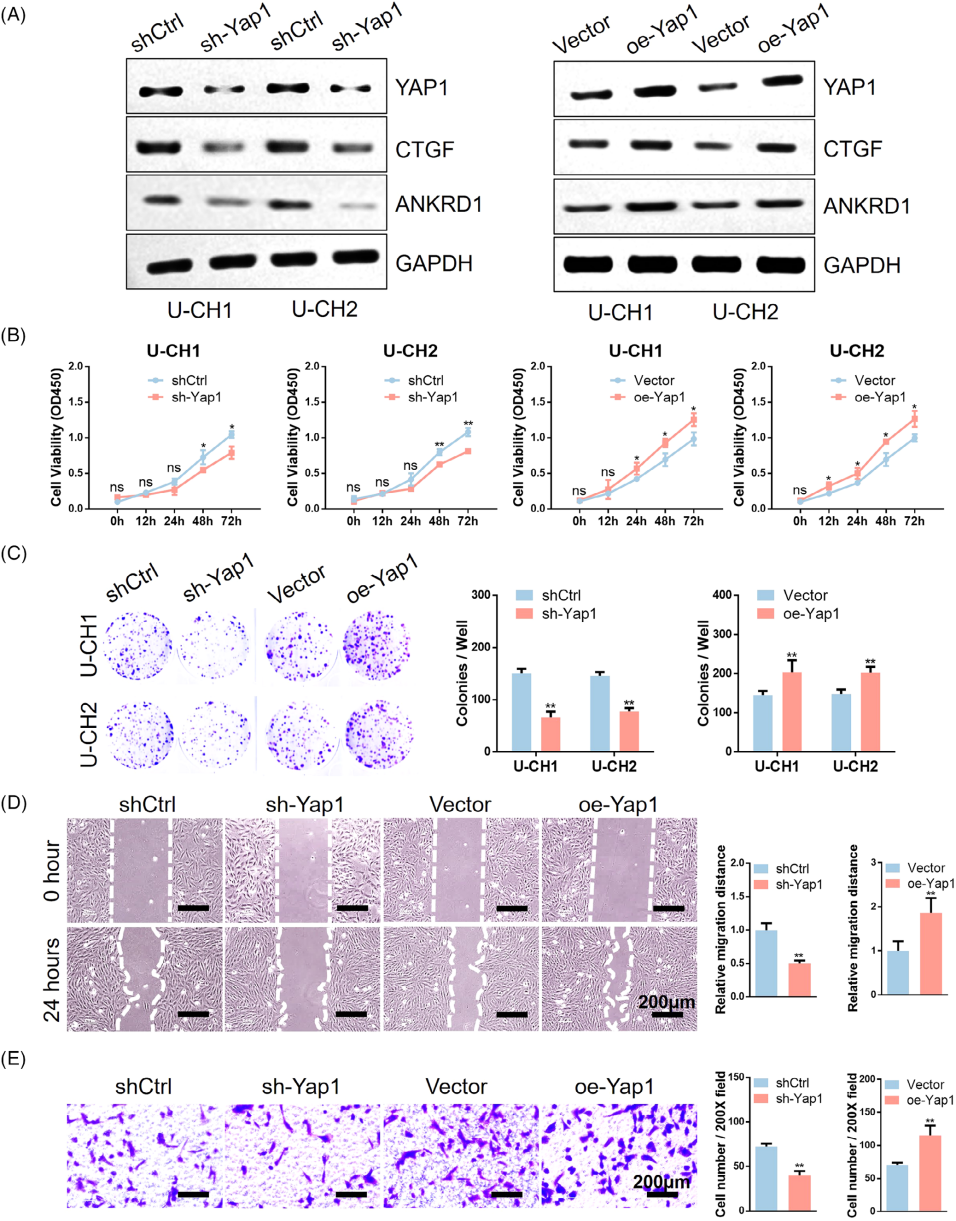
Yap1 promoted the proliferation and invasion of chordoma. (A) Left, WB analysis of YAP1 and downstream targets like CTGF and ANKRD1 in Yap1‐knockdown U‐CH1/U‐CH2 cells. Right, WB analysis of YAP1 and downstream targets like CTGF and ANKRD1 in Yap1‐overexpression U‐CH1/U‐CH2 cells. (B) CCK‐8 assays of knockdown and overexpression of Yap1 in U‐CH1/U‐CH2 cells. (C) Left, colony formation assays of knockdown and overexpression of Yap1 in U‐CH1/U‐CH2 cells. Right, quantitative analysis of colony formation assays. (D) Left, wound‐healing assays of knockdown and overexpression of Yap1 in U‐CH1 cells. Right, quantitative analysis of wound‐healing assays. (E) Left, Transwell assays of knockdown and overexpression of Yap1 in U‐CH1 cells. Right, quantitative analysis of Transwell assays.

Yap1, a key regulator of tissue growth and homeostasis, has been linked to several aspects of tumour stemness and growth. However, its direct role in chordoma remains unexplored. To address this aspect, this study generated shRNA targeting Yap1 (sh‐Yap1) and an overexpression plasmid for Yap1 (oe‐Yap1). Western blotting analysis confirmed the efficacy of sh‐Yap1 and oe‐Yap1 in chordoma cells, modulating YAP protein expression and its downstream targets (Figure 6A). Further assessments through colony formation and CCK‐8 assays unveiled the significant promotion of chordoma cell proliferation by Yap1 (Figure 6B,C). Wound healing assays demonstrated a significant increase in migration area after 24 h in the oe‐Yap1 group (Figure 6D and Figure S5A). Additionally, Transwell assays provided evidence that Yap1 plays a crucial role in enhancing invasiveness (Figure 6E and Figure S5B). Collectively, these findings establish the pivotal function of Yap1 in driving proliferation and invasion in chordoma.

### 2.7 CircTEAD1 promotes tumorigenesis of chordoma through the Hippo pathway

**FIGURE 7:**
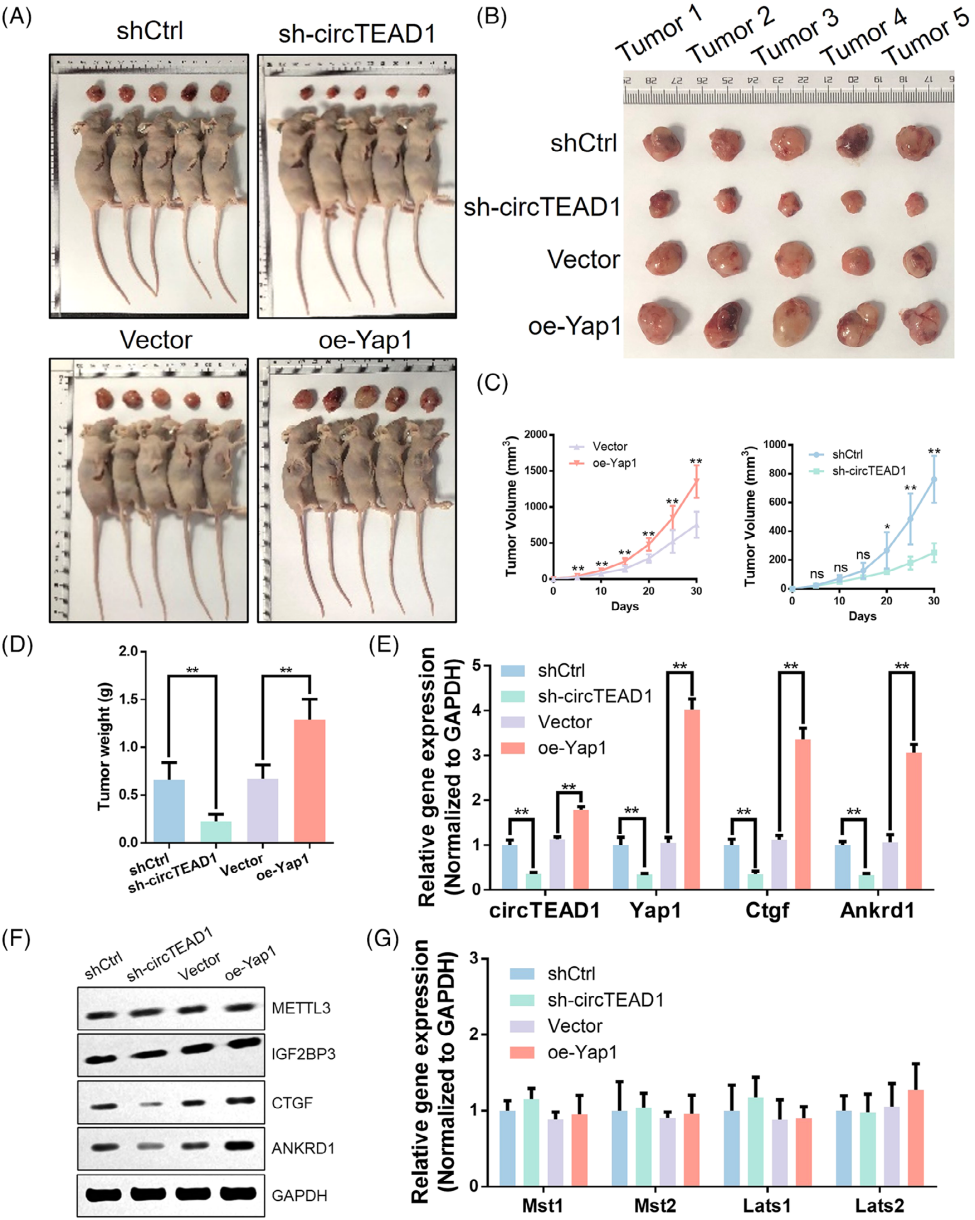
CircTEAD1 promotes tumorigenesis of chordoma through Hippo pathway. (A) U‐CH1 cells transfected with shCtrl, sh‐circTEAD1, empty vector and Yap1‐overexpression lentiviruses were percutaneously implanted into nude mice. (B) Tumours of each group were harvested and are exhibited. (C) Tumour growth curve. (D) Final tumour weights. (E) RT‐qPCR analysis of circTEAD1, Yap1, Ctgf and Ankrd1 in each group. (F) WB analysis of METTL3, IGF2BP3, CTGF and ANKRD1 in each group. (G) RT‐qPCR analysis of Mst1, Mst2, Lats1 and Lats2 in each group.

The relationship between the Hippo pathway and circTEAD1 was further investigated by establishing animal models using chordoma cells stably transfected with empty vector, shCtrl, sh‐circTEAD1 or oe‐Yap1. Remarkably, the knockdown of circTEAD1 led to a substantial reduction in tumour growth, while oe‐Yap1 notably accelerated it (Figure 7A−D). To explore if circTEAD1 influenced the levels of Yap1 and its downstream targets, further investigations were conducted. The findings depicted that sh‐circTEAD1 restrained the expression of both Yap1 and Ctgf or Ankrd1, whereas oe‐Yap1 reinforced their levels (Figure 7E). Importantly, Western blotting analyses across the different groups revealed that neither the downregulation of circTEAD1 nor the overexpression of Yap1 affected the expression of METTL3 and IGF2BP3 (Figure 7F). Meanwhile, the upstream targets of Yap1 were detected. The findings demonstrated that the alteration of circTEAD1 and Yap1 had no significant effect on MST and LATS pathways (Figure 7G). Moreover, these outcomes elucidate that circTEAD1‐dependent modulation of Yap1 is pivotal for tumorigenesis, suggesting a potential therapeutic target for chordoma management.

## 3 DISCUSSION

Chordomas primarily occur in the spine and sacral region,^18^ and sacral chordoma is characterized as an infiltrative and vessel‐rich tumour.^19^ Our previous work has confirmed that incomplete excision, invasion into the surrounding muscle, inadequate surgical margins and a higher level of tumour involvement are poor prognostic factors.^20^ Hence, further exploration of the molecular mechanism of chordoma development is warranted to identify potential therapeutic targets.^21^, ^22^

This study conducted genome‐wide identification of chordoma tissue, leading to the discovery and functional validation of circTEAD1. However, the role of circRNAs in chordoma remains relatively unknown. Only Lou et al. demonstrated that chordoma malignancy is augmented through a circTLK1/miR‐16‐5p/Smad3 positive feedback pathway.^23^ Similar to Lou et al., this study found that circTEAD1 is highly expressed in chordoma and promotes chordoma progression. However, contrary to the proposed mechanism, it was demonstrated that circTEAD1 could form a circTEAD1/IGF2BP3/Yap1 mRNA ternary complex in the cytoplasm to regulate chordoma proliferation and invasion through the Hippo signalling pathway. This contributes to the foundational knowledge of circRNA regulation in chordoma.

CircRNAs constitute a recently recognized category of ncRNAs characterized by their covalently closed loop structure.^24^ Recently, the involvement of circRNAs in malignant tumours has garnered increasing attention.^25^ At present, researchers have fully studied the mechanism by which circRNAs enact their biological functions. Among these mechanisms, the interaction between RBPs and circRNAs is a dynamic and diverse process,^26^ with circRNA‐protein complexes gaining particular attention.^27^ For instance, Du et al. discovered that the circFoxo3/Mdm2 complex prevents Foxo3 ubiquitination and degradation, thereby inhibiting cell apoptosis.^28^ Several studies have primarily focused on the formation of circRNA‐RBP complexes. For example, Chen et al. uncovered a cytoplasmic circNSUN2/IGF2BP2/HMGA2 RNA‐protein ternary complex that enhances the HMGA2 mRNA stability, thus facilitating colorectal liver metastasis.^29^ However, similar ternary complexes have been studied rarely.

The m6A modification is catalysed by m6A methyltransferases, including METTL3 and METTL14, among others, and is removed by m6A demethylases, ultimately recognized by reader proteins. METTL3, a key component among methyltransferase enzymes, plays an important role in regulating various tumours. Xiong et al. revealed that METTL3 mediated m6A modification on Jak1 mRNA in tumour‐infiltrating myeloid cells (TIMs), leading to immunosuppression of TIMs.^30^ This study attempted to confirm that the m6A modification of circTEAD1 is METTL3‐dependent and assessed whether it promotes circTEAD1 export to the cytoplasm. The localization of circRNA has been an important basis for its function. Huang et al. found that the nuclear regulatory proteins are often regulated by the URH49/UAP56 protein family and the lengths of mature circRNAs.^31^ Therefore, the function of circTEAD1 both in and out of the nucleus may be related to diverse regulatory mechanisms, which warrants further investigation.

The Hippo signalling pathway represents a common mechanism in tumorigenesis. YAP1 amplification is prevalent in cancers and is linked to cancer‐cell maintenance, cellular invasion, chemoresistance, hyperproliferation and metastasis.^32^ Recent scientific investigations have documented that circRNAs can modulate the activity of YAP1 through traditional mechanisms like competitive endogenous RNAs and peptide translation. Chen et al. demonstrated that the activation of YAP1 by m6A‐modified circCPSF6 contributes to the progression of malignancy in hepatocellular carcinoma.^33^ Additionally, Shah et al. highlighted brachyury as a pivotal regulator of stemness in chordoma, illustrating that this effect is achieved by governing the production and stability of YAP1.^17^

This study aimed to investigate whether a regulatory relationship exists between circRNAs and Yap1 in the context of chordoma, leading to the identification of circTEAD1. Interestingly, MS analysis demonstrated the interaction of circTEAD1 with IGF2BP3 and METTL3. IGF2BP3 could stabilize Yap1 mRNA by forming a ternary complex independent of m6A modification. Furthermore, this research revealed that the export of circTEAD1 to the cytoplasm is facilitated by METTL3 in an m6A methylation‐dependent manner. Furthermore, elevated circTEAD1 expression enhanced Yap1 mRNA stability by forming a circTEAD1/IGF2BP3/Yap1 ternary complex, which led to chordoma invasion and proliferation. Most notably, the findings present circTEAD1 as a promising therapeutic target, thus expanding the treatment options for human chordoma.

## 4 MATERIALS AND METHODS

### 4.1 Patients and tissue specimen collection

The investigation was performed on the tissue samples acquired from 20 chordoma patients who underwent surgical procedures. All participants submitted informed written consent, and the research was carried out in compliance with the principles defined in the Declaration of Helsinki. The ethical approval for the research protocols was granted by the Institutional Ethics Committee of the First Affiliated Hospital of Soochow University.

### 4.2 RNA sequencing

RNA sequencing was performed on three pairs of chordoma and matched adjacent normal tissue samples. The mRNA Seq sample preparation kit was utilized to construct the cDNA library. The Illumina sequence platform (Illumina) at General Biotechnology was employed to carry out sequencing. Differentially expressed circRNAs were identified on the criteria of fold change ≥ 2 and *p* < .05.

### 4.3 Cell culture

Human chordoma cell lines (U‐CH1/U‐CH2) were procured from ATCC. The IMDM‐RPMI media (4:1) containing 1% penicillin‑streptomycin stock solution and 10% foetal bovine serum were utilized to perform cell culture. The cultivation was maintained at 37˚C with 5% CO2 in a humidified cell incubator.

### 4.4 Cell proliferation assay

The cells (U‐CH1 and U‐CH2) were seeded on 96‐well plates (1 × 10^3^/well). After 24 h of culture, 10 µL of CCK‐8 reagent (FC101, TransGen) was added to the cells, and their viability was assessed by measuring the absorbance at OD450 at 12, 24, 48 and 72 h.

### 4.5 Colony formation assay

The cells (U‐CH1 and U‐CH2) in the logarithmic growth phase were harvested as cell suspensions. Then, they were placed into a 6‐well culture plate (1 × 10^3^/well). Dulbecco's modified eagle medium (DMEM) medium was utilized for cell culture, with medium replacement performed every 3 days. The cloning cultivation was terminated after continuous culture for 14 days. After that, the culture medium was removed, and cells underwent fixation for 20 min at room temperature with methanol. To facilitate fixation, .1% crystal violet was utilized to stain cell clones for 20 min. Photographs were captured to document and count the results of clone formation.

### 4.6 RNase R and Actinomycin D treatment

Following circTEAD1 identification, its stability was routinely tested using RNase R (Epicentre Technologies) and Actinomycin D (Sigma) according to their respective instructions.

### 4.7 RNA fluorescence in situ hybridization

General Biotechnology synthesized the oligonucleotide‐modified probe sequences for circTEAD1 and Yap1. The staining procedure followed the instructions of the RNA Fluorescence in situ Hybridization Kit (R0306M, Beyotime). Fluorescence microscopy (Zeiss LSM800) was employed for image acquisition. The probe sequences are provided below:

circTEAD1 FISH probe:

5′‐FITC‐TGAATCATTCCCGGCCAGAACCAGAATTTTACGAGGAAGA‐3′.

Yap1 mRNA FISH probe:

5′‐FITC‐TGCTGGCAGAGGTACATCATCAGGTATCTCAAAAGAAGAC‐3′.

### 4.8 Cytoplasmic and nuclear RNA extraction

The cytoplasmic and nuclear components were isolated by employing PARIS Kit (AM1921, Thermo Fisher). A cell fractionation buffer was used to perform lysis on an adequate number of chordoma cells (∼10^6^) for 5 min. Following 3 min of centrifugation, the final cytoplasmic and nuclear RNA were separated and obtained.

### 4.9 Cell transfection

Genelily Biotech Co., LTD designed and synthesized shRNAs and overexpression vectors. Vector (oe‐NC) is an overexpression of the empty control plasmid (pLV‐circ‐Puro for oe‐circTEAD1 and pLVX‐CMV‐Puro for oe‐Mettl3/Yap1). Transient transfection of siRNA and transfection of the overexpression vectors were performed utilizing Lipofectamine 3000 (Invitrogen, Thermo Fisher Scientific) as per the provided guidelines. The sequences are shown in Supporting Information (Table S1).

### 4.10 RT‐qPCR

Using the TRIzol reagent, RNA was isolated from all the specimens. Moreover, the concentration of RNA was quantified utilizing a NanoDrop 2000 spectrophotometer. Afterwards, cDNA was generated through reverse transcription from the extracted mRNA. After that, RT‐qPCR was carried out utilizing the ABI Step One Plus real‐time PCR system in combination with the SYBR Green RT‐PCR kit. Every specimen was examined in triplicate, and all steps were executed as per the provided instructions. The mRNA expression levels of the genes were normalized to the housekeeping gene Gapdh, and the 2^–ΔΔCt^ technique was utilized to evaluate them. A list of the primers used in this study can be found in Table S2.

### 4.11 RNA immunoprecipitation

The RIP experiments were conducted employing the Magna RIP Kit. As per the provided guidelines, magnetic beads were pre‐covered with antibodies (5 mg). These beads were then mixed with cell lysates, followed by the addition of proteinase K to break down proteins. Following the extraction of the complex that was precipitated from the beads, RNAs were isolated for RT‐qPCR analysis. The calculation of the relative enrichment was carried out by normalizing the input. The antibodies used in this study include METTL3 (86132S, CST), IGF2BP3 (86132S, CST), YAP (14074S, CST), GAPDH (HRP‐60004, Proteintech), CTGF (25474‐1‐AP, Proteintech) and ANKRD1 (11427‐1‐AP, Proteintech).

### 4.12 Methylated RNA immunoprecipitation

In order to evaluate the levels of m6A in circRNA, MeRIP assays were carried out using the Magna MeRIP m6A Kit from Millipore. Magnetic beads were combined with either 10 mg of m6A antibody or IgG and then utilized for RNA sample immunoprecipitation. The primers used for MeRIP‐qPCR analysis were generated through SRAMP software (http://www.cuilab.cn/sramp), with sequences mentioned below:

forward primer—GTCAGATTTATGACAAATTT,
reverse primer—GGCTTGACGTCTTGTGAGGA.

The relative m6A enrichment was normalized against the input.

### 4.13 Silver staining and MS analysis

The Fast Silver Stain Kit (P0017S, Beyotime) was utilized to perform silver staining, as per the provided protocol. Proteome Discoverer software (version 1.4; Thermo Fisher Scientific) was utilized to perform protein identification and quantification. Genelily Biotech Co., LTD conducted the MS analysis. A protein was considered significant if it had a ratio (circTEAD1/CTRL) greater than 2 and a minimum of 2 unique peptides (Table S3).

### 4.14 RNA pull‐down

Probes labelled with biotin targeting the circTEAD1 junction positions were created by General Biotechnology in Anhui, China. The Pierce Magnetic RNA‐Protein Pull‐Down Kit from Thermo Fisher Scientific was utilized for carrying out the RNA pull‐down procedure as per the provided guidelines. The sequences of the probe pool for circTEAD1 are as follows:

5′‐Biotin‐CTGTTTGAATCATTCCCGGCCAGAACCAGAATTTTACGAGGAAGAAGGCA‐3′.

Probe for NC:

Biotin‐5′‐GACTTGAAGCCTAGCCCGATCGACTGGGATTTAGCAGCATAAATAGCCGC‐3.

### 4.15 Western blotting analysis

The protein lysates were applied onto SDS‐PAGE gels and then transferred to a polyvinylidene difluoride (PVDF) membrane (Millipore). After incubating with primary antibodies overnight at 4°C, the membranes were hybridized with secondary antibodies at room temperature for 1 h. Subsequently, the electrochemiluminescence reagent (NCM Biotech) was utilized to observe the results.

### 4.16 Luciferase reporter assay

The sequence of wild type (WT) and Mutant Yap1 mRNA was synthesized and cloned downstream of the p‐GLO Dual‐Luciferase vector (Vigenebio). Twenty‐four hours prior to transfection with 1 µg p‐GLO Dual‐Luciferase reporter (p‐GLO‐WT/Mut‐Yap1), U‐CH1 and U‐CH2 cells with established circTEAD1 stable knockdown or over‐expression were placed in 24‐well dishes at a density of 30% confluency/well. After 48 h, the relative luciferase activity was assessed by determining the ratio between Firefly and Renilla luciferase activities employing a dual‐luciferase reporter assay system (Promega). Rellina luciferase activity served as an internal control. In the circTEAD1 overexpression or knockdown group, the relative Luc/Rluc ratio was normalized to that of the control sample.

### 4.17 Transwell assays

Invasion assays were carried out by seeding 2 × 10^5^ cells in 500 µL of serum‐free medium into transwell chambers containing 8‐µm pore membranes. After the incubation period of 48 h, the cells that had moved underwent fixation, while the cells on top were delicately removed. The migrating cells were then dyed using crystal violet and counted using a microscope. Each trial was repeated three times.

### 4.18 Invasion assays

A total of 200 000 cells were seeded in transwell chambers with 8‐µm pore filters coated by Matrigel. The cells that invaded the membrane were stained and photographed. The cells were placed in 12‐well dishes for the wound healing experiment. Once they reached 90% confluency, a 10 µL plastic pipette tip was used to create scratch wounds. The percentage of migrated areas was quantified using Image J software.

### 4.19 Animal models

Stably transfected U‐CH1 cells (1×10^7^ cells/mouse) were subcutaneously injected into either side of the armpit regions of 8‐week‐old male Balb/c nude mice for tumorigenicity studies. The examination and measurement of tumour volumes were carried out every 5 days by employing the equation mentioned below:

volume = .5 × length × width^2^.

After 30 days post‐injection, the mice were euthanized, and the subcutaneous growth of each tumour was assessed.

### 4.20 Statistical analyses

Experiments were conducted at least thrice, with data from one illustrative trial displayed as mean ± S.D. The significance of variances was determined utilizing a two‐way ANOVA or a two‐tailed Student's *t*‐test. Evaluation of CDFS was carried out employing the Kaplan−Meier technique, with the Log‐rank test facilitating the comparison. Statistical significance was established at *p* < .05.

## AUTHOR CONTRIBUTIONS

Hanwen Li, Yingchuang Tang and Xingbang Ruan contributed equally to this work.

## CONFLICT OF INTEREST STATEMENT

All authors declare no conflict of interest.

## ETHICS APPROVAL AND CONSENT TO PARTICIPATE

The study protocols received ethical approval from the Institutional Ethics Committee of the First Affiliated Hospital of Soochow University.

## Supporting information


Figure S1 Characteristics of circTEAD1
A. RT‐qPCR analysis for the 17 upregulated circRNAs related to Hippo pathway in normal and chordoma tissues.
B. RT‐qPCR analysis for the expressions of circTEAD1 and TEAD1 treated with RNase R in U‐CH2 cells.
C. RT‐qPCR analysis for the expressions of circTEAD1 and TEAD1 treated with actinomycin D in U‐CH2 cells after 0, 4, 8, 12 and 24 hours.
D. RT‐qPCR analysis for cytoplasmic and nuclear mRNA fractionation experiment. β‐actin and U3 were applied as positive controls in the cytoplasm and nucleus, respectively.
E. RNA fluorescence in situ hybridization (FISH) for circTEAD1 in U‐CH2 cells. Nuclei were stained with DAPI.

Figure S2 The influence of circTEAD1 m6A modification
A. RT‐qPCR of cytoplasmic and nuclear mRNA fractionation experiment after U‐CH1/U‐CH2 treated with wild‐type or mutant‐type of circTEAD1.
B. Colony formation assays after overexpressing mutant‐type of circTEAD1 in U‐CH1/U‐CH2 cells.
C. Transwell assays after overexpressing mutant‐type of circTEAD1 in U‐CH1/U‐CH2 cells.
D. Wound‐healing assays after overexpressing mutant‐type of circTEAD1 in U‐CH1/U‐CH2 cells.

Figure S3 METTL3 facilitates cytoplasmic export of m6A‐modified circTEAD1 for functional activity
A. RT‐qPCR of cytoplasmic and nuclear mRNA fractionation experiment after U‐CH2 treated with knockdown or expression of Mettl3.
B. RNA‐FISH of circTEAD1 in U‐CH2 after treated with knockdown or expression of METTL3.

Figure S4 Correlation between circTEAD1 and Hippo signaling
A. Relative quantitative analysis of Fig. 5C and D.
B. Luciferase mRNA expression of luciferase reporter gene with Yap1‐WT or Yap1‐Mut in control and circTEAD1‐knockdown U‐CH1/U‐CH2 cells.
C. Luciferase mRNA expression of luciferase reporter gene with Yap1‐WT or Yap1‐Mut in control and circTEAD1‐overexpression U‐CH1/U‐CH2 cells.
D. RIP assays showing the association of Yap1 and upstream targets of Hippo‐pathway with circTEAD1 in U‐CH2 cells.
E. RIP assay showing the association of IGF2BP3 with Yap1 in IGF2BP3‐knockdown U‐CH2 cells.

Figure S5 Yap1 promoted the proliferation and invasion of chordoma
A. Left, wound‐healing assays of knockdown and overexpression of Yap1 in U‐CH2 cells. Right, quantitative analysis of wound‐healing assays.
B. Left, Transwell assays of knockdown and overexpression of Yap1 in U‐CH2 cells. Right, quantitative analysis of Transwell assays.

**Table S1** Target sequences of shRNAs and overexpression plasmid used in this study.

**Table S2** Sequences of primers used in this work.

**Table S3** List of top 15 candidates of circTEAD1‐interacting proteins that were identified by RNA pull down and MS.

## Data Availability

The authors will supply the relevant data in response to reasonable requests. Additional data are made available in supporting information.
