# Supplementary figures and images for: N6‐methyladenosine‐modified circTEAD1 stabilizes Yap1 mRNA to promote chordoma tumorigenesis
Source: Clin Transl Med. 2024 Apr 24;14(4):e1658. doi: 10.1002/ctm2.1658 (PMC11043093; doi:10.1002/ctm2.1658)

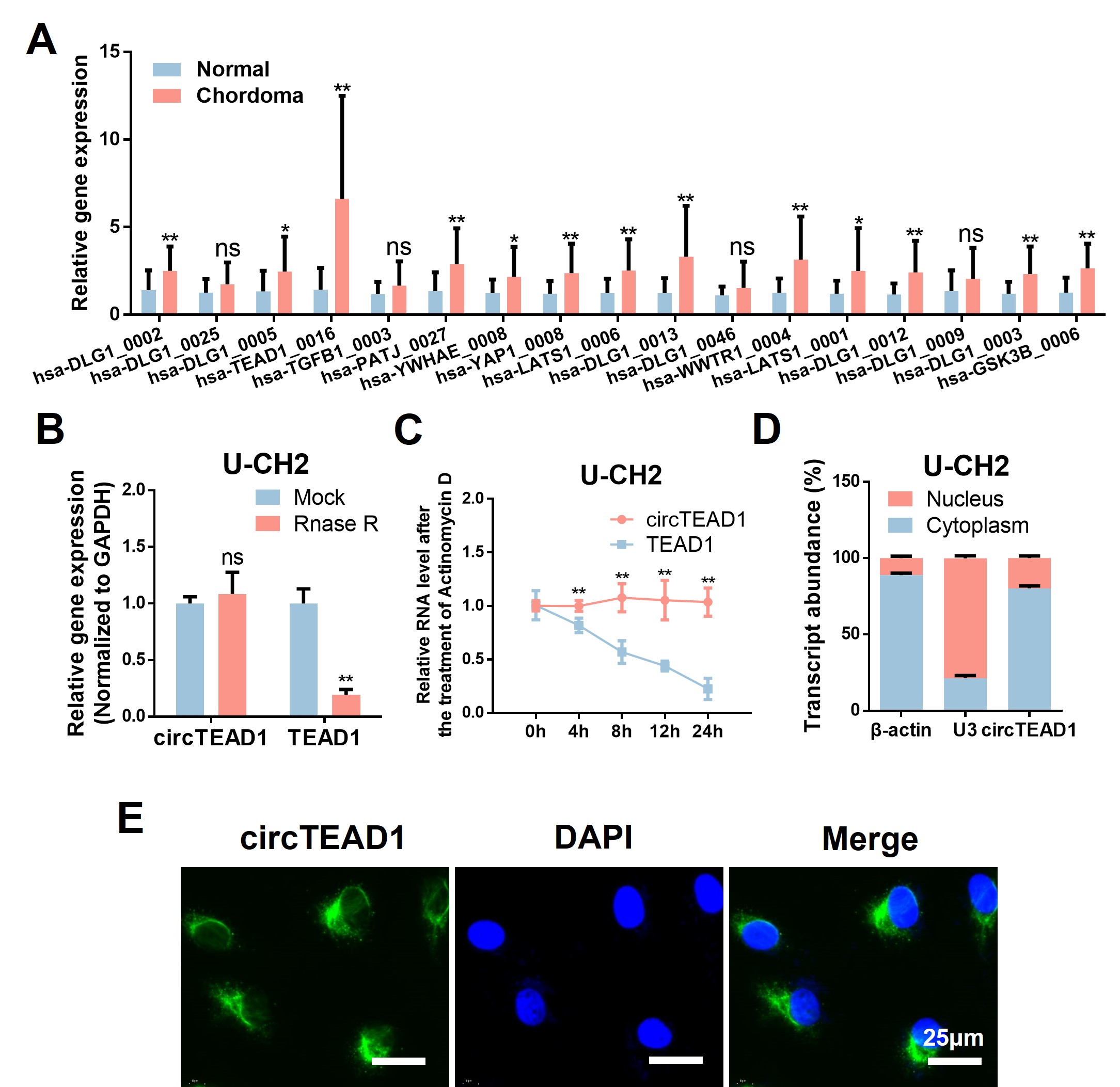

Supplement: Supplementary file 1 — Figure S1 Characteristics of circTEAD1 A. RT‐qPCR analysis for the 17 upregulated circRNAs related to Hippo pathway in normal and chordoma tissues. B. RT‐qPCR analysis for the expressions of circTEAD1 and TEAD1 treated with RNase R in U‐CH2 cells. C. RT‐qPCR analysis for the expressions of circTEAD1 and TEAD1 treated with actinomycin D in U‐CH2 cells after 0, 4, 8, 12 and 24 hours. D. RT‐qPCR analysis for cytoplasmic and nuclear mRNA fractionation experiment. β‐actin and U3 were applied as positive controls in the cytoplasm and nucleus, respectively. E. RNA fluorescence in situ hybridization (FISH) for circTEAD1 in U‐CH2 cells. Nuclei were stained with DAPI. [file CTM2-14-e1658-s008.jpg]

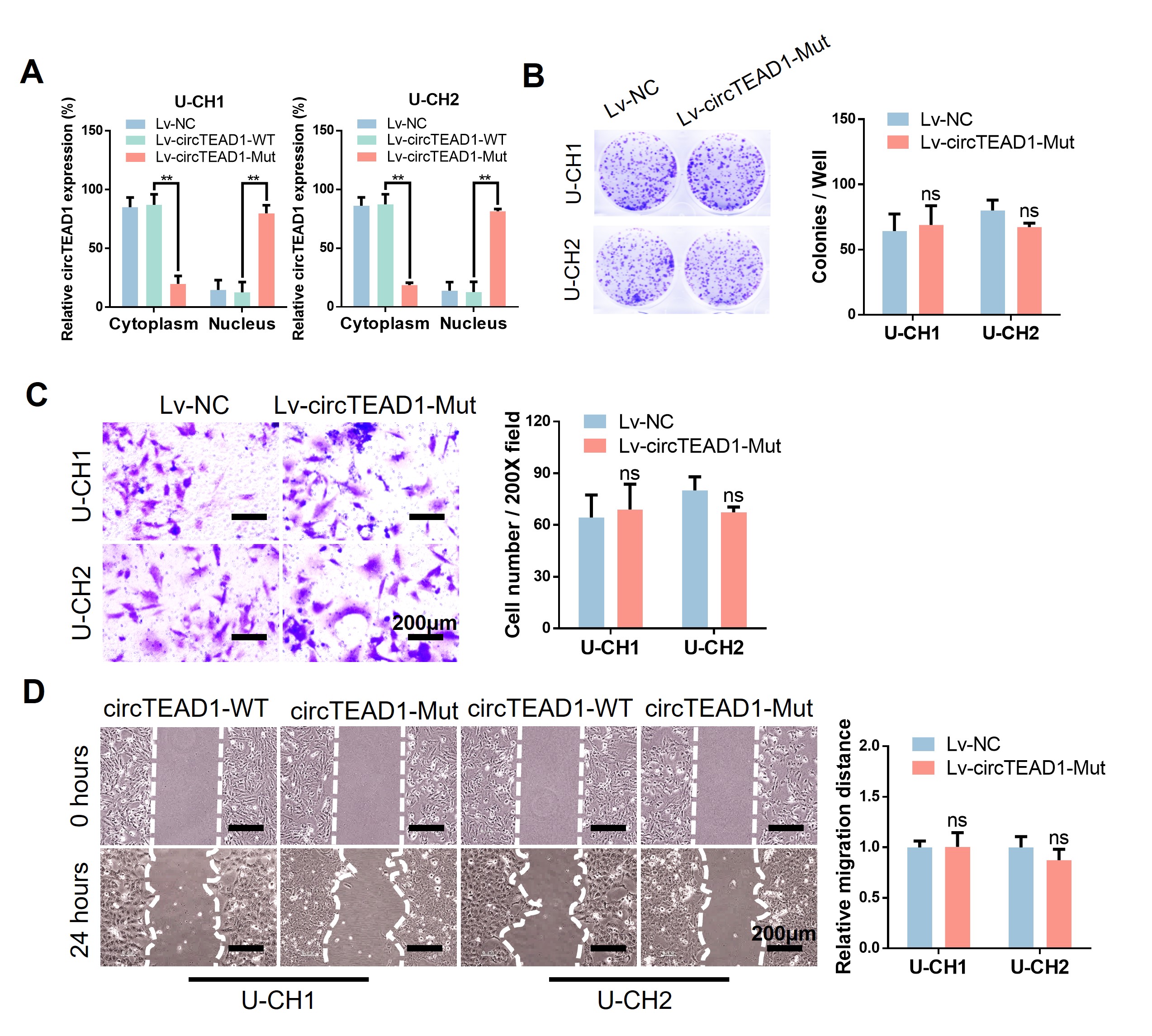

Supplement: Supplementary file 2 — Figure S2 The influence of circTEAD1 m6A modification A. RT‐qPCR of cytoplasmic and nuclear mRNA fractionation experiment after U‐CH1/U‐CH2 treated with wild‐type or mutant‐type of circTEAD1. B. Colony formation assays after overexpressing mutant‐type of circTEAD1 in U‐CH1/U‐CH2 cells. C. Transwell assays after overexpressing mutant‐type of circTEAD1 in U‐CH1/U‐CH2 cells. D. Wound‐healing assays after overexpressing mutant‐type of circTEAD1 in U‐CH1/U‐CH2 cells. [file CTM2-14-e1658-s002.jpg]

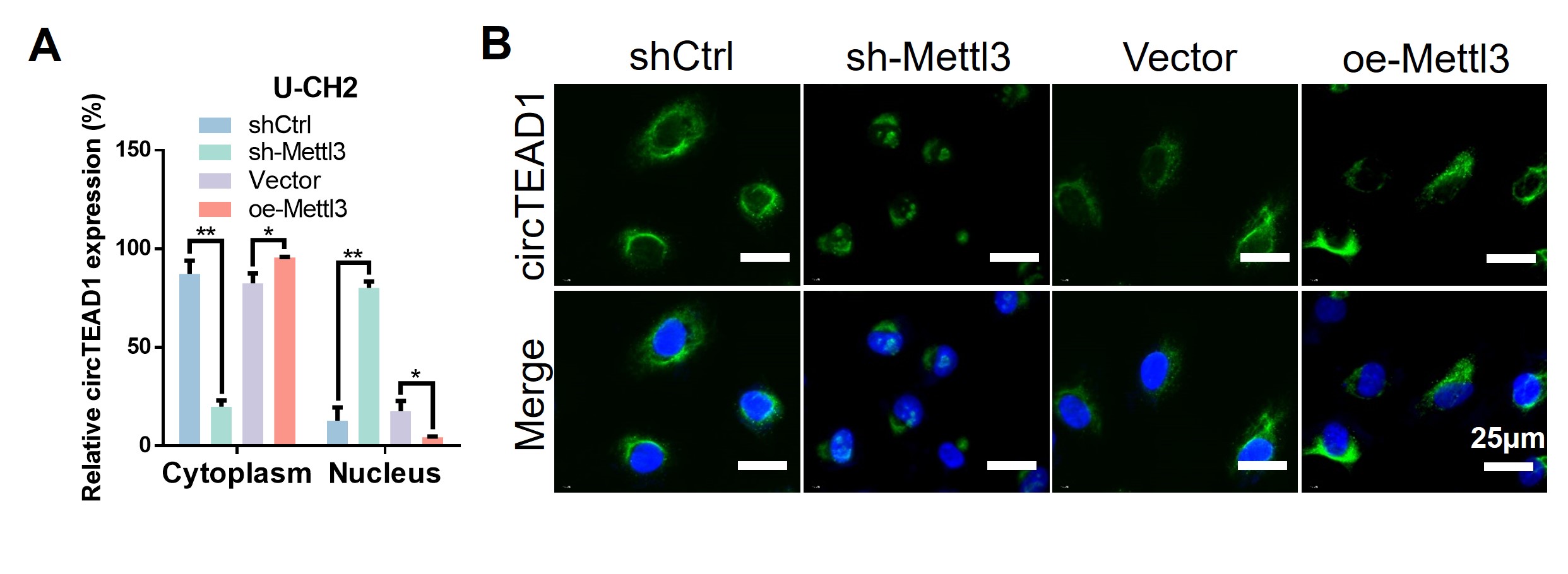

Supplement: Supplementary file 3 — Figure S3 METTL3 facilitates cytoplasmic export of m6A‐modified circTEAD1 for functional activity A. RT‐qPCR of cytoplasmic and nuclear mRNA fractionation experiment after U‐CH2 treated with knockdown or expression of Mettl3. B. RNA‐FISH of circTEAD1 in U‐CH2 after treated with knockdown or expression of METTL3. [file CTM2-14-e1658-s007.jpg]

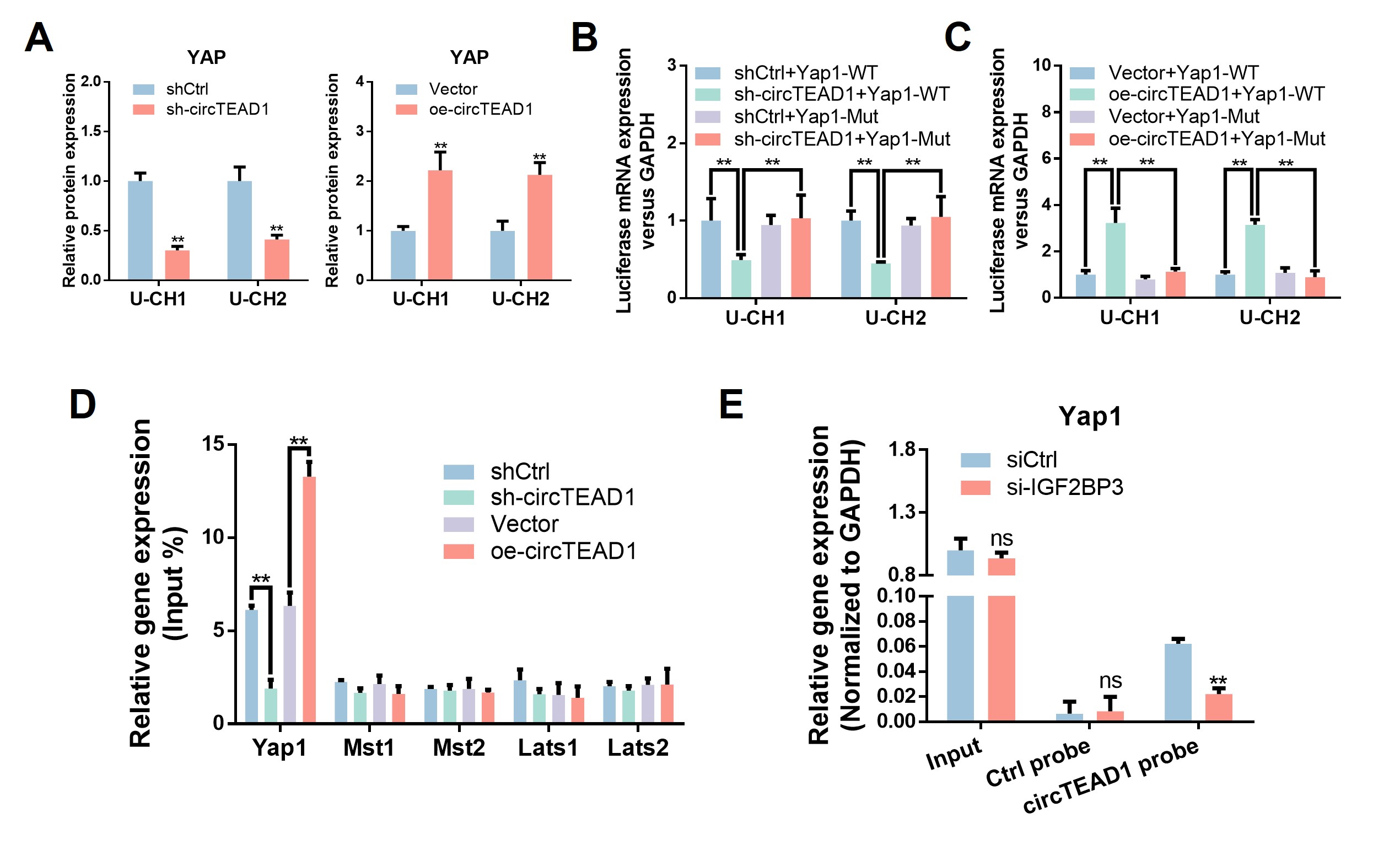

Supplement: Supplementary file 4 — Figure S4 Correlation between circTEAD1 and Hippo signaling A. Relative quantitative analysis of Fig. 5C and D. B. Luciferase mRNA expression of luciferase reporter gene with Yap1‐WT or Yap1‐Mut in control and circTEAD1‐knockdown U‐CH1/U‐CH2 cells. C. Luciferase mRNA expression of luciferase reporter gene with Yap1‐WT or Yap1‐Mut in control and circTEAD1‐overexpression U‐CH1/U‐CH2 cells. D. RIP assays showing the association of Yap1 and upstream targets of Hippo‐pathway with circTEAD1 in U‐CH2 cells. E. RIP assay showing the association of IGF2BP3 with Yap1 in IGF2BP3‐knockdown U‐CH2 cells. [file CTM2-14-e1658-s001.jpg]

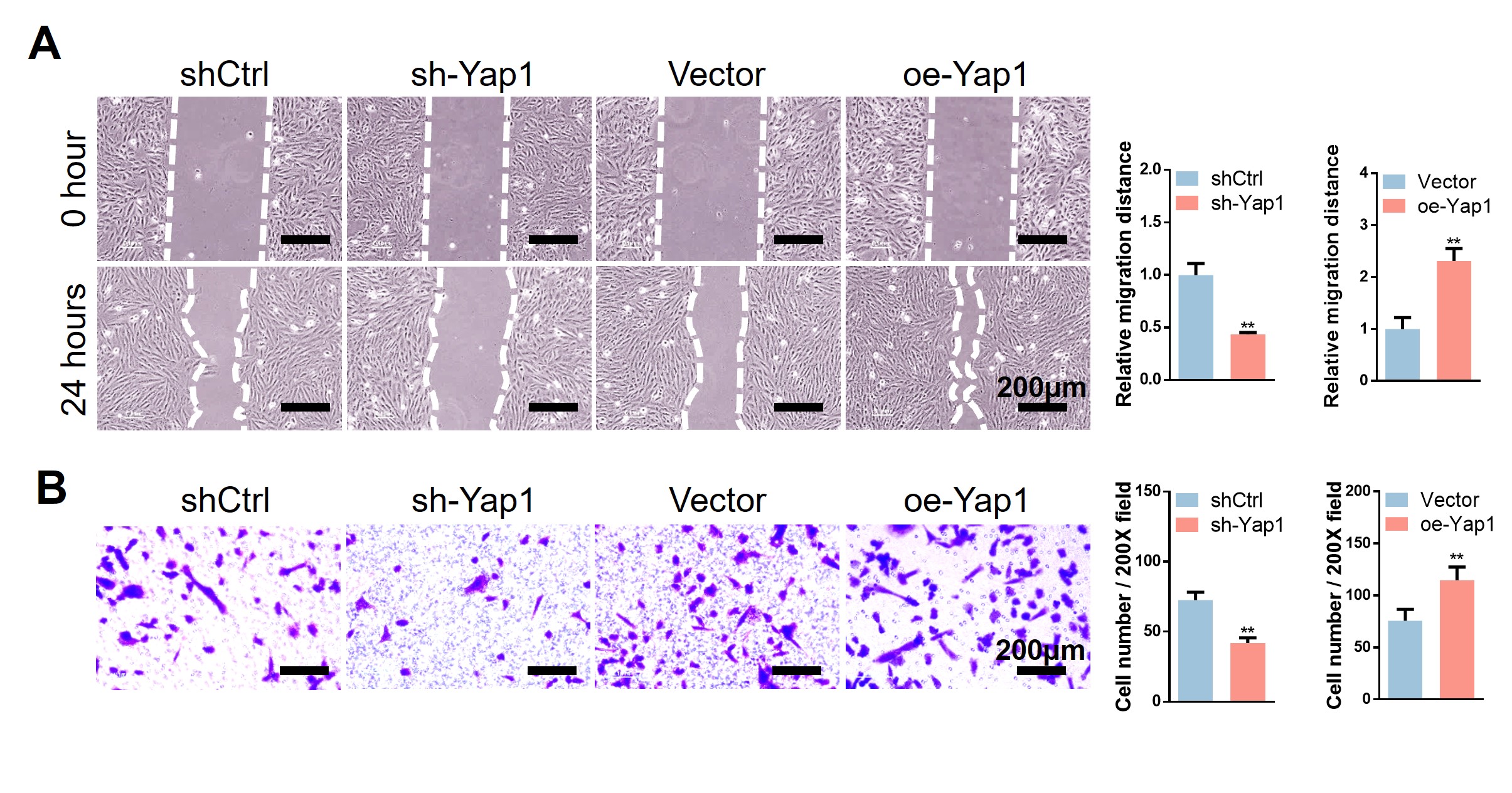

Supplement: Supplementary file 5 — Figure S5 Yap1 promoted the proliferation and invasion of chordoma A. Left, wound‐healing assays of knockdown and overexpression of Yap1 in U‐CH2 cells. Right, quantitative analysis of wound‐healing assays. B. Left, Transwell assays of knockdown and overexpression of Yap1 in U‐CH2 cells. Right, quantitative analysis of Transwell assays. [file CTM2-14-e1658-s004.jpg]
