# Supplementary material for: N6‐methyladenosine‐modified circTEAD1 stabilizes Yap1 mRNA to promote chordoma tumorigenesis
Source: Clin Transl Med. 2024 Apr 24;14(4):e1658. doi: 10.1002/ctm2.1658 (PMC11043093; doi:10.1002/ctm2.1658)
Supplement: Supplementary file 6 — Table S1 Target sequences of shRNAs and overexpression plasmid used in this study. [file CTM2-14-e1658-s005.docx]

**Table S1.** Target sequences of shRNAs and overexpression plasmid used in this study.

| shCtrl | ACGCTTAATAGCATTAGGCATGTTCAAGAGACATGCCTAATGCTATTAAGTTTTTTT |
| --- | --- |
| sh-circTEAD1 | AAAATTCTGGTTCTGGCCGGTTCAAGAGACCGGCCAGAACCAGAATTTTTTTTTT |
| oe-circTEAD1 | TTCTGGCCGGGAATGATTCAAACAGGGCAGCCAGGATCCTCACAAGACGTCAAGCCTTTTGTGCAGCAGGCCTACCCCATCCAGCCAGCGGTCACAGCCCCCATTCCAGTACAACAAACACCTCTTCGTGCACATTGGGCATGCCAACCATTCTTACAGTGACCCATTGCTTGAATCAGTGGACATTCGTCAGATTTATGACAAATTTCCTGAAAAGAAAGGTGGCTTAAAGGAACTGTTTGGAAAGGGCCCTCAAAATGCCTTCTTCCTCGTAAAATTCTGG |
| sh-Mettl3 | CAGGAGATCCTAGAGCTATTAAATATTCAAGAGATATTTAATAGCTCTAGGATCTCCTGTTTTTT |
| oe-Mettl3 | ATGTCGGACACGTGGAGCTCTATCCAGGCCCACAAGAAGCAGCTGGACTCTCTGCGGGAGAGGCTGCAGCGGAGGCGGAAGCAGGACTCGGGGCACTTGGATCTACGGAATCCAGAGGCAGCATTGTCTCCAACCTTCCGTAGTGACAGCCCAGTGCCTACTGCACCCACCTCTGGTGGCCCTAAGCCCAGCACAGCTTCAGCAGTTCCTGAATTAGCTACAGATCCTGAGTTAGAGAAGAAGTTGCTACACCACCTCTCTGATCTGGCCTTAACATTGCCCACTGATGCTGTGTCCATCTGTCTTGCCATCTCCACGCCAGATGCTCCTGCCACTCAAGATGGGGTAGAAAGCCTCCTGCAGAAGTTTGCAGCTCAGGAGTTGATTGAGGTAAAGCGAGGTCTCCTACAAGATGATGCACATCCTACTCTTGTAACCTATGCTGACCATTCCAAGCTCTCTGCCATGATGGGTGCTGTGGCAGAAAAGAAGGGCCCTGGGGAGGTAGCAGGGACTGTCACAGGGCAGAAGCGGCGTGCAGAACAGGACTCGACTACAGTAGCTGCCTTTGCCAGTTCGTTAGTCTCTGGTCTGAACTCTTCAGCATCGGAACCAGCAAAGGAGCCAGCCAAGAAATCAAGGAAACATGCTGCCTCAGATGTTGATCTGGAGATAGAGAGCCTTCTGAACCAACAGTCCACTAAGGAACAACAGAGCAAGAAGGTCAGTCAGGAGATCCTAGAGCTATTAAATACTACAACAGCCAAGGAACAATCCATTGTTGAAAAATTTCGCTCTCGAGGTCGGGCCCAAGTGCAAGAATTCTGTGACTATGGAACCAAGGAGGAGTGCATGAAAGCCAGTGATGCTGATCGACCCTGTCGCAAGCTGCACTTCAGACGAATTATCAATAAACACACTGATGAGTCTTTAGGTGACTGCTCTTTCCTTAATACATGTTTCCACATGGATACCTGCAAGTATGTTCACTATGAAATTGATGCTTGCATGGATTCTGAGGCCCCTGGCAGCAAAGACCACACGCCAAGCCAGGAGCTTGCTCTTACACAGAGTGTCGGAGGTGATTCCAGTGCAGACCGACTCTTCCCACCTCAGTGGATCTGTTGTGATATCCGCTACCTGGACGTCAGTATCTTGGGCAAGTTTGCAGTTGTGATGGCTGACCCACCCTGGGATATTCACATGGAACTGCCCTATGGGACCCTGACAGATGATGAGATGCGCAGGCTCAACATACCCGTACTACAGGATGATGGCTTTCTCTTCCTCTGGGTCACAGGCAGGGCCATGGAGTTGGGGAGAGAATGTCTAAACCTCTGGGGGTATGAACGGGTAGATGAAATTATTTGGGTGAAGACAAATCAACTGCAACGCATCATTCGGACAGGCCGTACAGGTCACTGGTTGAACCATGGGAAGGAACACTGCTTGGTTGGTGTCAAAGGAAATCCCCAAGGCTTCAACCAGGGTCTGGATTGTGATGTGATCGTAGCTGAGGTTCGTTCCACCAGTCATAAACCAGATGAAATCTATGGCATGATTGAAAGACTATCTCCTGGCACTCGCAAGATTGAGTTATTTGGACGACCACACAATGTGCAACCCAACTGGATCACCCTTGGAAACCAACTGGATGGGATCCACCTACTAGACCCAGATGTGGTTGCACGGTTCAAGCAAAGGTACCCAGATGGTATCATCTCTAAACCTAAGAATTTATAG |
| sh-Yap1 | CACCAGTGCAGCAGAATATTTCAAGAGAATATTCTGCTGCACTGGTGTTTTTT |
| oe-Yap1 | ATGGATCCCGGGCAGCAGCCGCCGCCTCAACCGGCCCCCCAGGGCCAAGGGCAGCCGCCTTCGCAGCCCCCGCAGGGGCAGGGCCCGCCGTCCGGACCCGGGCAACCGGCACCCGCGGCGACCCAGGCGGCGCCGCAGGCACCCCCCGCCGGGCATCAGATCGTGCACGTCCGCGGGGACTCGGAGACCGACCTGGAGGCGCTCTTCAACGCCGTCATGAACCCCAAGACGGCCAACGTGCCCCAGACCGTGCCCATGAGGCTCCGGAAGCTGCCCGACTCCTTCTTCAAGCCGCCGGAGCCCAAATCCCACTCCCGACAGGCCAGTACTGATGCAGGCACTGCAGGAGCCCTGACTCCACAGCATGTTCGAGCTCATTCCTCTCCAGCTTCTCTGCAGTTGGGAGCTGTTTCTCCTGGGACACTGACCCCCACTGGAGTAGTCTCTGGCCCAGCAGCTACACCCACAGCTCAGCATCTTCGACAGTCTTCTTTTGAGATACCTGATGATGTACCTCTGCCAGCAGGTTGGGAGATGGCAAAGACATCTTCTGGTCAGAGATACTTCTTAAATCACATCGATCAGACAACAACATGGCAGGACCCCAGGAAGGCCATGCTGTCCCAGATGAACGTCACAGCCCCCACCAGTCCACCAGTGCAGCAGAATATGATGAACTCGGCTTCAGGTCCTCTTCCTGATGGATGGGAACAAGCCATGACTCAGGATGGAGAAATTTACTATATAAACCATAAGAACAAGACCACCTCTTGGCTAGACCCAAGGCTTGACCCTCGTTTTGCCATGAACCAGAGAATCAGTCAGAGTGCTCCAGTGAAACAGCCACCACCCCTGGCTCCCCAGAGCCCACAGGGAGGCGTCATGGGTGGCAGCAACTCCAACCAGCAGCAACAGATGCGACTGCAGCAACTGCAGATGGAGAAGGAGAGGCTGCGGCTGAAACAGCAAGAACTGCTTCGGCAGGCAATGCGGAATATCAATCCCAGCACAGCAAATTCTCCAAAATGTCAGGAGTTAGCCCTGCGTAGCCAGTTACCAACACTGGAGCAGGATGGTGGGACTCAAAATCCAGTGTCTTCTCCCGGGATGTCTCAGGAATTGAGAACAATGACGACCAATAGCTCAGATCCTTTCCTTAACAGTGGCACCTATCACTCTCGAGATGAGAGTACAGACAGTGGACTAAGCATGAGCAGCTACAGTGTCCCTCGAACCCCAGATGACTTCCTGAACAGTGTGGATGAGATGGATACAGGTGATACTATCAACCAAAGCACCCTGCCCTCACAGCAGAACCGTTTCCCAGACTACCTTGAAGCCATTCCTGGGACAAATGTGGACCTTGGAACACTGGAAGGAGATGGAATGAACATAGAAGGAGAGGAGCTGATGCCAAGTCTGCAGGAAGCTTTGAGTTCTGACATCCTTAATGACATGGAGTCTGTTTTGGCTGCCACCAAGCTAGATAAAGAAAGCTTTCTTACATGGTTATAG |
