# Supplementary material for: N6‐methyladenosine‐modified circTEAD1 stabilizes Yap1 mRNA to promote chordoma tumorigenesis
Source: Clin Transl Med. 2024 Apr 24;14(4):e1658. doi: 10.1002/ctm2.1658 (PMC11043093; doi:10.1002/ctm2.1658)
Supplement: Supplementary file 7 — Table S2 Sequences of primers used in this work. [file CTM2-14-e1658-s003.docx]

**Table S2.** Sequences of primers used in this work.

| Gene | Forward primer (5’-3’) | Reverse primer (5’-3’) |
| --- | --- | --- |
| Yap1 | CCACCAGTCCACCAGTGCAG | GCAGTTGCTGCAGTCGCATC |
| Mst1 | TCAGGCTCCCAGCTTGTCCT | TAGTCACCCTCACAGGCCCC |
| Mst2 | GTGTGGAGAGTGTGGGCACC | ACCATGGTCCCCAAGTCGGA |
| Lats1 | TGTTGTCCCTGCTGGCACTG | TTTCATGCCCACTGCTCGGG |
| Lats2 | TCTGGAAATAGCCGGCAGCG | TGGGGTGGCTCTCATCTGCT |
| Tead1 | TTTGTGCAGCAGGCCTACCC | CCAATGGAGCGACCTTGCCA |
| β-actin | GAGCTACGAGCTGCCTGACG | TGCCAGGGCAGTGATCTCCT |
| U3 | AGAAGCCACAAGGAGCAGGC | TGGCGAATGGTACTTGCAGGG |
| Ctgf | GTGTACCGCAGCGGAGAGTC | CACGGTTTGGTCCTTGGGCT |
| Ankrd1 | GCTCGCCTTAGTCGTCACCC | GCGCCGAAGTTGCATTCCAG |
| hsa-DLG1_0002 | CTCAGAAAAGGAGATCGTATTATATCGCTT | CTGGAGATGCTGGTGTCTGG |
| hsa-DLG1_0025 | TTCAATTCTAAAACGAGAGATAAAGGGCTTC | CTGGAGATGCTGGTGTCTGG |
| hsa-DLG1_0005 | TCAGAAAAGGAGATCGTATTATATCGGCAA | ACGTAAGTTGGTGTTTCCAAGC |
| hsa-TEAD1_0016 | CTTGAATCAGTGGACATTCG | ACTGGAATGGGGGCTGTGAC |
| hsa-TGFB1_0003 | CACCAACTATTGCTTCAGGGGA | CCGGTAGTGAACCCGTTGAT |
