# Supplementary material for: N6‐methyladenosine‐modified circTEAD1 stabilizes Yap1 mRNA to promote chordoma tumorigenesis
Source: Clin Transl Med. 2024 Apr 24;14(4):e1658. doi: 10.1002/ctm2.1658 (PMC11043093; doi:10.1002/ctm2.1658)
Supplement: Supplementary file 8 — Table S3 List of top 15 candidates of circTEAD1‐interacting proteins that were identified by RNA pull down and MS. [file CTM2-14-e1658-s006.docx]

**Table S3. List of top 15 candidates of circTEAD1-interacting proteins that were identified by RNA pull down and MS**

| **Gene Name** | **Unique peptides** | **Unique sequence coverage [%]** | **Mw(kDa)** | **Accession** |
| --- | --- | --- | --- | --- |
| IGF2BP3 | 176 | 57.5 | 63.7 | O00425 |
| EIF3A | 37 | 28.5 | 166.5 | Q14152 |
| EPRS1 | 50 | 46.2 | 170.5 | P07814 |
| METTL3 | 85 | 49.4 | 64.5 | Q86U44 |
| ACTN4 | 35 | 47.2 | 104.8 | O43707 |
| PYGB | 37 | 49.3 | 96.6 | P11216 |
| SPTAN1 | 93 | 49.8 | 284.5 | Q13813 |
| MYH9 | 62 | 39.1 | 226.5 | P35579 |
| SNRNP200 | 46 | 30.2 | 244.5 | O75643 |
| CLTC | 46 | 37.6 | 191.6 | Q00610 |
| HSP90B1 | 44 | 56.9 | 92.4 | P14625 |
| VIM | 37 | 75.5 | 53.6 | P08670 |
| EEF2 | 41 | 56.4 | 95.3 | P13639 |
| ENO1 | 24 | 61.1 | 47.1 | P06733 |
| LMNA | 44 | 66.3 | 74.1 | P02545 |
